# Identification of Gas Channeling and Construction of a Gel-Enhanced Foam Plugging System for Oxygen-Reduced Air Flooding in the Changqing Oilfield

**DOI:** 10.3390/gels8060373

**Published:** 2022-06-13

**Authors:** Tengfei Wang, Liangliang Wang, Haoliang Qin, Cong Zhao, Zongxian Bai, Xingbang Meng

**Affiliations:** 1 School of Petroleum Engineering, China University of Petroleum (East China), Qingdao 266580, China; wangtengfei@upc.edu.cn (T.W.); qinhl1999@163.com (H.Q.); mengxingbang@163.com (X.M.); 2 Research Institute of Shaanxi Yanchang Petroleum (Group) Co., Ltd., Xi’an 710065, China; zhaocongycyjy@163.com; 3 Operation Area of Nanpu Oilfield, Jidong Oilfield Company of CNPC, Tangshan 063200, China; baizongxian77@163.com

**Keywords:** gas channeling channels, oxygen-reduced air flooding, foam plugging, fuzzy membership function, controlling factors

## Abstract

The accurate identification of gas channeling channels during foam-assisted oxygen-reduced air flooding (FAORAF) and the analysis of the main controlling factors are essential to propose reasonable and effective countermeasures to enhance oil recovery (EOR). However, there are few comprehensive studies on identifying gas channeling channels, the influencing factors, and the corresponding plugging EOR systems in FAORAF. The channeling channels of the injection and production wells of the Changqing Oilfield, China, under varying development schemes are identified utilizing fuzzy membership function theory in this work to obtain their primary distribution. The characteristics and influence factors of gas channeling channels are analyzed by numerical simulation using CMG. The recovery performance of each foam blocking system is evaluated by twin-tube sand pack models. As well, based on the features of reservoir fractures, a new gel-enhanced foam plugging system is developed. The results show that channeling channels chiefly develop along NE 60–70° and that foam could reduce gas channeling. Natural and artificial fractures are the principal factors causing gas channeling, followed by the injection method and gas injection rate. Under the premise of the injection and migration efficiency, the optimal gel system is a 0.1% HPAM + 0.1% organic chromium crosslinking agent. The addition of gel increases the viscosity of the liquid phase and strengthens the mechanical strength of the foam liquid film. At a permeability ratio of 12, the recovery factors of the binary plugging systems composed of microspheres, PEG, and gel combined with foam are 40.89%, 45.85%, and 53.33%, respectively. The movable gel foam system has a short breaking time (only 18 days) and a recovery factor of about 40% at a permeability ratio of 20. To be suitable for oil reservoirs with microfractures, an improved ternary gel foam system—0.1% HPAM + 0.1% chromium crosslinking agent + 0.05–0.1% nano-SiO_2_—is developed. Compared with the binary gel foam system, the recovery rate of the new nano-SiO_2_ gel foam system after 15 days of ageing using the core splitting test is 25.24% during the FAORAF process, increasing by 12.38%.

## 1. Introduction

Low and ultra-low permeability light oil reservoirs have tiny pore throats, and water flooding development has long faced major problems such as the failure to inject and recover [1,2]. The air flooding technique has obvious advantages such as easy injection, sufficient gas sources, and environmental protection [3,4]. Air flooding has succeeded in oil reservoirs with relatively homogeneous formation properties, with significant economic benefits. For continental sedimentary reservoirs with severe heterogeneity, the risk of gas channeling is greater [5,6]. In addition, to prevent explosions and ensure the safety and controllability of air flooding, light oil reservoirs are usually flooded with oxygen-reduced air. Oxygen-reduced air flooding (ORAF) is a development method in which deoxidized air whose volume ratio of oxygen is reduced to less than 10% is injected into oil reservoirs to displace crude oil by utilizing deoxidized air generation and pressurized injection systems [7,8,9]. Besides maintaining the reservoir pressure, the low-temperature oxidation heat generation, flue gas flooding, and swelling effect can be fully utilized to enhance oil recovery (EOR) during the ORAF process.

Due to the strong anisotropy of the oil reservoirs and the development of microfractures, the channeling channels are easily formed by long-term water flooding. Compared to water flooding and ORAF, foam-assisted oxygen-reducing air flooding (FAORAF) has a significant effect on the low/ultra-low permeability oil reservoirs enhancing oil recovery (EOR). Foam has a sound sealing ability, but gas channeling with varying degrees is still prone to occur because of the unsatisfactory sealing ability and stability of the foam system, which greatly reduces the oil recovery efficiency of FAORAF [10,11,12,13]. Thus, the accurate identification of gas channeling channels during FAORAF and the analysis of the main controlling factors are essential to propose reasonable and effective countermeasures to recover residual oil.

In recent years, numerous researchers have given great attention to relevant research on identifying water/gas channeling channels to provide the basis for putting forward reasonable development methods. The common channeling channel identification methods mainly include the pressure index (PI) and dimensionless pressure index (DPI) based on the well test, the inter-well tracer test results, and the comprehensive identification of dynamic parameters [14]. The PI and DPI method could qualitatively determine whether there is a channeling channel based on a single wellhead pressure drop curve. However, it has a higher cost and dramatic errors. For inter-well tracer technology, the tracer is injected, and the existence of the channeling channel is judged by the tracer output status of the production wells [15]. The static geological and production dynamic information, combined with the fuzzy mathematical theory—the fuzzy comprehensive evaluation technique—has realized a qualitatively identified and quantitatively described channeling channel [16].

Due to the traditional method only being able to evaluate the influence of a single factor on the seepage channel, a new evaluation model with geological and production parameters and seepage directions for seepage channels in the gas–liquid two-phase flow process was proposed [17]. To identify the types of central flow channels in complex porous media, Li et al. (2019) established a mathematical model based on the assumption of equivalent flow, proposed a classification method of main flow channels, and realized quantitative characterization [18]. Liu et al. (2021) took three formations of Cretaceous carbonate reservoirs in the Central and Southern Mesopotamian Basin, identified and located channeling channels by the amount of water injection and liquid production, and studied the characteristics and causes of channeling channels [19]. The channeling flow between injection and production wells results in a large amount of residual oil in the oil reservoir, and the water cut continues to rise. Xu et al. (2021) recognized and classified the channeling channels for the North Buzazi heavy oil reservoir using the fuzzy comprehensive evaluation, the production-injection profile, and the PI method. The identification results have good consistency among the three methods [20].

Numerous indoor research and on-site applications show that gas channeling occurs in the process of FAORAF, which is mainly affected by the channeling channel generated in the water flooding stage, the gas injection method, and the injection speed [21]. The channeling channel is effectively blocked and combined with the reasonable injection speed and mode to ensure the successful application of FAORAF. The influencing factors of gas channeling are generally studied through indoor physical simulation experiments and numerical simulations. In this study, based on the reservoir geological characteristics, combined with the identification results and distribution trends of the channeling channels, the Computer Modelling Group (CMG) numerical simulation was used to study the gas channeling characteristics and their main controlling factors.

Based on quantitatively identifying channeling channels and recognizing their main controlling factors, the development of anti-channeling blocking agents is essential to stimulate oil reservoir recovery. Plugging agent-based oil recovery processes include a big family of EOR systems, such as a single-type plugging agent (microsphere, foam, gel), a binary compound-type plugging agent (microsphere-foam, gel-foam, elastic gel particles (PEG)-foam), and an emerging ternary compound plugging agent (particle-gel-PEG, particle-gel-microsphere) [22,23,24,25]. The last two techniques generally receive great interest in laboratory research because of their widespread field application and significant economic benefits. Jia et al. (2019) pointed out that the foam stability prepared by adding viscous polymers or polymers and a single crosslinking agent alone (crosslinking method) is minimal. Through double cross-linking with chromium III acetate (Cr^3+^) and polyethyleneimine (PEI), the in situ generation method of foamed gel was explored to improve the stability of the foam. The foamed gel has good sealing performance, and the gas permeability recovery rate reaches 93.90% [26]. The reaction of the polymer and crosslinker in low temperature (<50 °C) reservoirs is incredibly prolonged. To this end, Qi et al. (2018) developed a foam-gel system capable of spontaneously generating in situ heat. When the ambient temperature is fixed at 30 °C and the pH is 6.8, the gelation time is 40 h, the gel strength reaches the G level, and the volume expansion rate at 10 MPa exceeds 130%, which has a significant volume expansion and water blocking capacity [27]. The water channeling and gas channeling of microfractured reservoirs have always limited their efficient development. Wang et al. (2020) carried out experiments on interface properties, particle adsorption effects, and rheological properties, revealing the stabilization mechanism of fly ash on three-phase foams [28]. The study indicated that the foam system composed of 0.5 wt% AOS + 4 wt% suspending agent + 8 wt% fly ash had good stability.

A concise review of the above works shows that most studies are limited to identifying the water/gas channeling channel using varying methods. Still, the quantitative analysis of the characteristics and controlling factors of gas channeling during ORAF is rarely documented. It is worth noting that, based on the identification of channeling channels and their developing laws, few scholars have conducted more in-depth research on the EOR scheme applicable to the target reservoir. The fuzzy comprehensive evaluation theory combined with tracer test results is used to identify the channeling channels in the water flooding and the FAORAF stages of the Changqing Oilfield, China. The gas–oil ratio, oil production rate, and N_2_ mole fraction of the produced gas are analyzed by numerical simulation, and the main controlling factors of the gas channeling channel are defined. The recovery performance of each foam blocking system is evaluated by twin-tube sand pack models. Based on the characteristics of the reservoir fracture, the new gel-enhanced foam plugging system is developed. The research results could guide the quantitative identification of channeling channels and the analysis of controlling factors and propose reasonable countermeasures to promote the oilfield application of the FAORAF technique.

## 2. Methods and Experiments

### 2.1. Numerical Simulation

**Figure 1:**
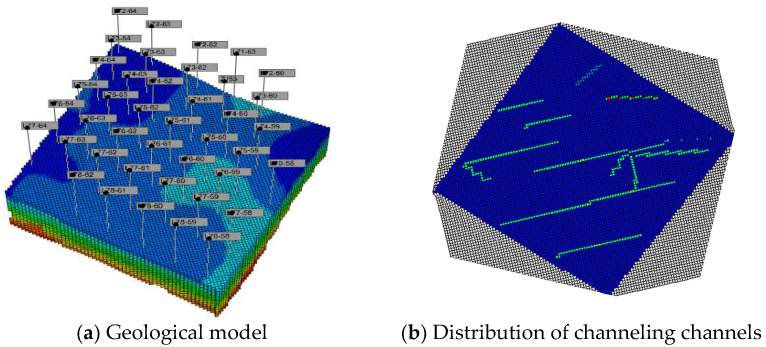
Geological model and distribution of channeling channels in the test area.

**Figure 2:**
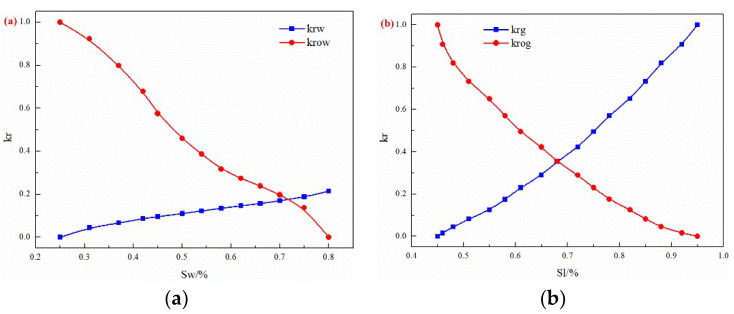
The phase permeability: (**a**) oil and water, (**b**) gas and liquid.

Based on the oil reservoir basic characteristics, a 3D geological model is established, and the gas–oil ratio, oil production rate, and N_2_ mole fraction of the produced gas are analyzed by numerical simulation. The channeling characteristics of water flooding and gas flooding are quantitatively clarified. The components flowing in the water flooding development stage are oil, water, and dissolved gas. During the EOR of the gas injection stage, the flow components may include N_2_, surfactants, and foam. There are 31 production wells and 13 water injection wells in the model. The corner grid system is adopted, and the grid system (Nx × Ny × Nz) is 118 × 107 × 12. The geological model and the channeling channel of the test area are shown in Figure 1. The channeling channels are represented in high-permeability strips in the geological model. The oil reservoir pressure is 12.5 MPa, and the rock compressibility is 2 × 10^−5^ kPa^−1^. The phase permeability curves are shown in Figure 2.

### 2.2. Materials and Methods

The light crude oil used in the first experiment came from the Changqing Oilfield, China, and its viscosity is 2 mPa·s (55 °C). The formation water salinity composition is shown in Table 1. The particles, PEG, hydrolyzed polyacryamide (HPAM), and chromium cross-linking agents were all provided by the Changqing Oilfield. The chemicals such as n-hexane, the foaming agent, and nano-SiO_2_ used in the experiments were purchased from Damao Chemical Reagent Factory, Tianjin.

### 2.3. Static Performance Evaluation Experiment of the Plugging System

1. Original particle size

First, 0.5 g of the plugging agent sample was added to the sealing system, and then 100 mL of n-hexane was added as the dispersion medium. After the mixed solution was stirred by a magnetic stirrer for 10 min, it was placed in an ultrasonic cleaner for ultrasonic dispersion for 5 min. The dispersed sample was injected into the sample cell with a rubber tip dropper and put into the groove of the instrument. The median initial particle size (D50) of the sample was measured by a laser particle size analyzer (Malvern Panalytical) with white oil as the dispersion medium.

2. Hydration expansion performance

A high-temperature and high-pressure reactor were used to simulate the oil reservoir conditions, and the microspheres were hydrated and expanded for 5, 10, 20, 30, and 55 days. The particle size distribution of the polymer microspheres after different hydration times is measured by a laser particle size analyzer.

3. Gelation time

Gelatin systems of different concentrations were prepared using formation water, HPAM, and chromium cross-linking agents. The prepared solution was sealed in a stoppered graduated cylinder and placed in a constant temperature water bath to form a gel at a constant temperature of 55 °C. By visual inspection, the tongue length of the gel was observed to judge its strength intuitively. The gel formation time is generally defined as the time it takes for the gel to deform down to about half of the position, usually with the sample bottle upside down.

4. Evaluation indexes

The gel strength was quantitatively evaluated by the breakthrough vacuum method. A total of 15 mL of organic chromium gel systems with different concentrations was prepared and placed at the same temperature (55 °C) for ageing after the gelation. The water precipitation and strength of the gel system were measured at 7, 15, and 30 days of ageing, and the stability of the gel system was evaluated. The storage and loss modulus of the ordinary and strengthened gel foams were measured by a rheometer (MCR301).

### 2.4. EOR Performance Evaluation Experiment of the Plugging System

1. Binary foam plugging system

Through the identification of channeling channels and the analysis of the influencing factors of gas channeling, it was found that the FAORAF technique can effectively prevent gas channeling. Still, there are also serious cases of gas channeling. However, the effect of using a single blocking agent to avoid gas channeling is not very satisfactory, so it is necessary to use the blocking agent to strengthen the foam for anti-gas channeling. For the microsphere, PEG, and gels-reinforced foam, the anti-gas channeling performance evaluation experiment was carried out. The experiment used a twin-tube model of the sand pack (60 cm × φ 2.5 cm), and the investigation was carried out at a temperature of 55 °C and at a backpressure of 12 MPa. Table 2 shows the parameters of the EOR physical simulation experiment of the sealing channeling system. Gas flooding was carried out at 0.5 mL·min^−1^, and the oil output and displacement pressure difference were recorded every time 0.1 PV gas was injected. The binary foam plugging system was injected at a rate of 0.1 mL·min^−1^, with a gas–liquid ratio of 1:1, and the gas flooding was carried out at a rate of 0.5 mL·min^−1^ to calculate the oil recovery factor.

2. Enhanced nano-SiO_2_ gel foam system

After 15 days of ageing (55 °C, 12 MPa), the plugging recovering performances of the gel foam and the newly improved nano-SiO_2_ gel foam system are comparatively studied. To simulate the existence of microfractures, the artificial core was split from the middle (9.0 cm × φ 2.5 cm). The porosities are 0.25 and 0.21, and the permeability before and after the core splitting is 6.5 × 10^−3^ μm^2^ and 165 × 10^−3^ μm^2^, respectively.

## 3. Results and Discussion

### 3.1. Identification of Channeling Channels

**Figure 3:**
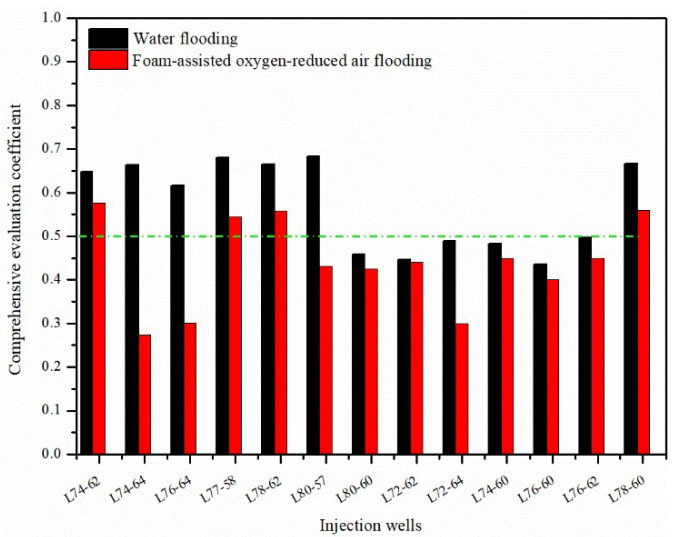
Identification results of the channeling channels of injection wells in the water flooding and FAORAF stages.

Generally, the standard value of the comprehensive evaluation coefficient of 0.50 is selected as the limit for judging whether the channeling channel is developed [20]. For ascending the half trapezoid model, the larger the comprehensive evaluation coefficient value, the greater the possibility of the channeling channels existing in the oil reservoir around the injection and the production well groups during the oilfield development process. Figure 3 shows the comprehensive identification results of injection wells in the water flooding and FAORAF stages, respectively. During water flooding, it was found that eight injection wells, including L74-62, L74-64, etc., were more likely to develop channeling channels. However, the five injection well groups—L72-62, L72-64, etc.—are less likely to develop channeling channels. Compared to water flooding, the comprehensive evaluation coefficient of all the injection wells decreased after the FAORAF, verifying that the FAORAF technology could reduce the degree of channeling. This is because foam has a practical channel plugging ability that reduces the channeling of the displacement medium in gas flooding [12].

**Figure 4:**
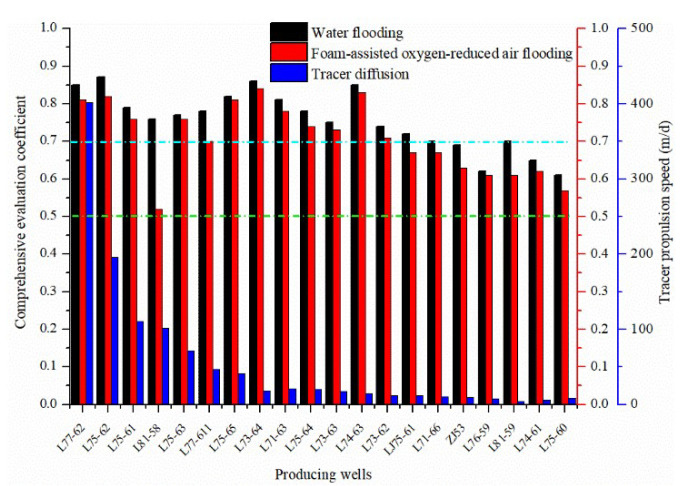
Identification results of the channeling channels of the production wells in the water flooding and FAORAF stages.

Furthermore, as seen in Figure 4, the comprehensive evaluation coefficients of all the production wells are greater than 0.5—some even exceed 0.7—and the production wells are more likely to develop channeling channels, which is in good agreement with the tracer test results. Similar to the identification results of the injection wells, the comprehensive evaluation coefficients of the production wells in the water flooding are larger than those in the FAORAF stage. Additionally, although the FAORAF can effectively inhibit the development of channeling channels after the FAORAF is carried out, the possibility of developing strong channeling channels is still high in 13 production wells such as L77-62. The reservoir is soaked by the injected water for a long time, and the formation strength is reduced. Higher gas injection and gas production easily disturb and damage the formation, resulting in ubiquitous and varying degrees of gas channelings. Based on the comprehensive evaluation and the tracer monitoring results, a classification standard for the comprehensive identification of channeling channels by fuzzy mathematics in production wells was established. The *F_Z_* < 0.5, 0.5 < *F_Z_* < 0.7, and *F_Z_* > 0.7 mean that there is an undeveloped, a weak, and a strong channeling channel, respectively. The FAORAF technique reduced the channeling in the production wells, but most of the wells still had channeling channels. Therefore, in the development of the plugging system, the combination of plugging agents and foam can be considered to prevent channeling.

### 3.2. Comprehensive Evaluation Theory of Fuzzy Mathematics

The identification method of the fuzzy mathematics comprehensive evaluation coefficient selects the static and dynamic indicators that affect the development of the channeling channel, uses the variation coefficient theory to calculate the weight value of each static and dynamic index, and finally obtains the quantitatively comprehensive evaluation coefficient. The method comprehensively utilizes various geological and production parameters and dynamic monitoring results in the process of oilfield development and has the advantages of high accuracy and low cost [14,16].

1. The coefficient of variation method calculates the weight
  - The coefficient of variation of each indicator
    $$V_{i}=\frac{\sigma_{i}}{\bar{x_{i}}}(i=1,2,3\cdots n)$$
  - The weight of each index
    $$\omega_{i}=\frac{V_{i}}{\sum_{i=1}^{n}V_{i}}$$
2. The trapezoidal model calculates the membership
  - Mathematical model of the ascending half trapezoid
    If the larger the value of the evaluation index is, the more likely the channeling channel is to exist, then the ascending half trapezoidal distribution model is used to calculate its membership degree.
    $$r_{ij}=\frac{x_{ij}-x_{i\min}}{x_{i\max}-x_{i\min}}$$
  - Mathematical model of the descending half trapezoid
    $$r_{ij}=\frac{x_{i\max}-x_{ij}}{x_{i\max}-x_{i\min}}$$

3. Comprehensive evaluation coefficient

The membership degree of each dynamic and static evaluation index, $r_{ij}$, is multiplied by its weight value, $\omega_{i}$, and then summed and recorded as $F_{Z}$, which is the comprehensive discriminant parameter of the dynamic and static dominant channels of each injection well and production well.

$$F_{J}=\sum_{i=1}^{n_{1}}\left(r_{Ji}\times \omega_{i}\right)\;F_{S}=\sum_{i=1}^{n_{2}}\left(r_{Si}\times \omega_{i}\right)\;F_{Z}=F_{J}\omega_{J}+F_{S}\omega_{S}$$

4. Evaluation indexes

The evaluation index of the fuzzy comprehensive discrimination of the channeling channels mainly comes from two aspects. The static geological indicators easily cause channeling channels, and dynamic indicators can represent the characteristics of oilfield production. The evaluation index system of fuzzy comprehensive identification generally includes geological static parameters of the injection and production well groups (permeability, porosity, and effective thickness), the production dynamics information of the injection wells (injection rate, cumulative injection per unit thickness, injection times, etc.), and the production dynamics indicators of the production wells (water cut, rate of daily fluid increase, production times, etc.). The coefficients of variation of porosity, permeability, and effective thickness are 0.02, 0.26, and 0.29, respectively, and the values of the weights are 0.03, 0.46, and 0.51, respectively.

### 3.3. The Characteristics of Gas Channeling and Controlling Factors

**Figure 5:**
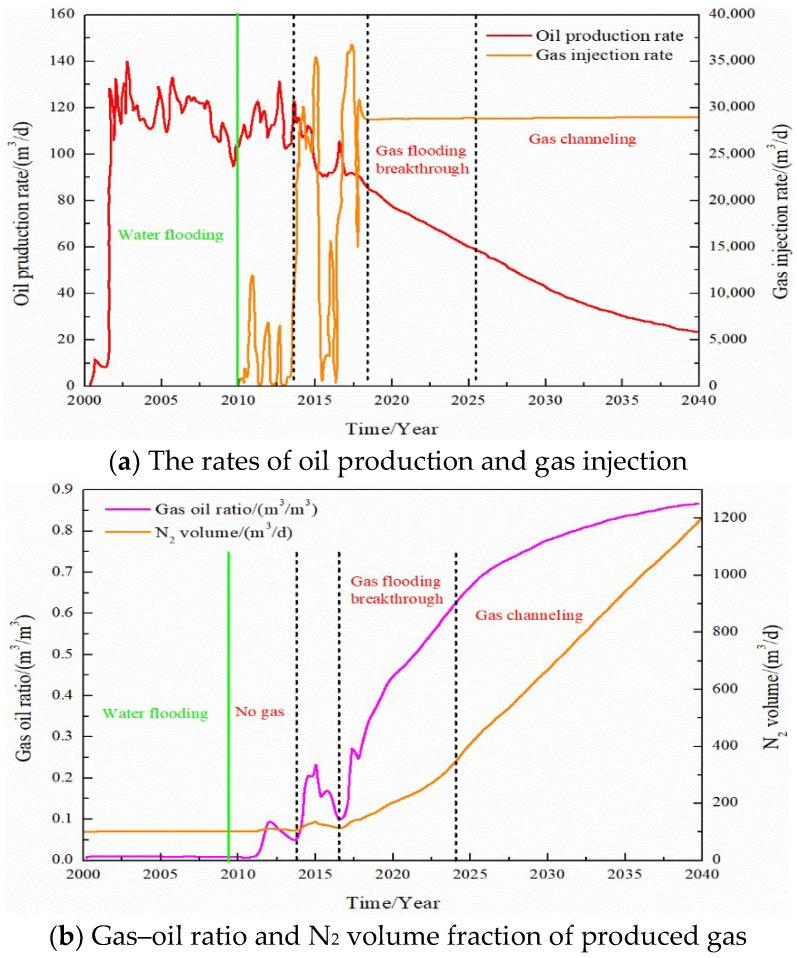
Comparison of dynamic production curves.

**Figure 6:**
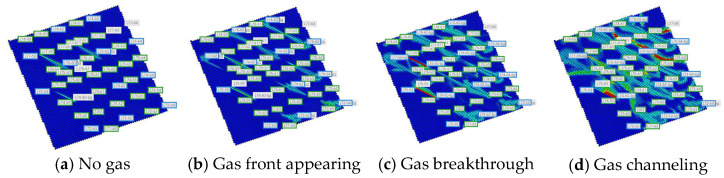
Saturation field of N_2_ content.

In the process of simulating gas injection development, the corresponding relationship between the oil production rate, gas–oil ratio, and N_2_ volume in the produced gas is used to study the gas channeling characteristics of gas flooding, as shown in Figure 5. In the process of oxygen reduction air flooding, the N_2_ content saturation field in the reservoirs at different stages is shown in Figure 6. Combining the above, the classification standard of gas channeling is divided. The classification standard of gas channeling in FAORAF is shown in Table 3. 

The oil production rate increased rapidly in the initial water injection stage and then began to fluctuate and decrease. After this stage, the gas injection development began, the oil production rate showed an upward trend, the production gas–oil ratio did not change, and the amount of N_2_ produced was very small, indicating that the gas injection development was effective without gas channeling. With the increase in the gas injection time and gas injection rate, the oil production rate began to show a downward trend. The content of produced N_2_ fluctuated greatly, and the gas flooding front appeared. With the continued gas injection, the oil production rate dropped rapidly, the N_2_ content of the produced gas increased rapidly, and the gas flooding broke through. Continued gas injection will cause gas channeling, the content of produced N_2_ will increase, and the oil production rate will continue to decline. Gas channeling tends to occur when the N_2_ mole fraction of the produced gas is more significant than 60% and the gas–oil ratio is higher than 400 m^3^·t^−1^.

**Figure 7:**
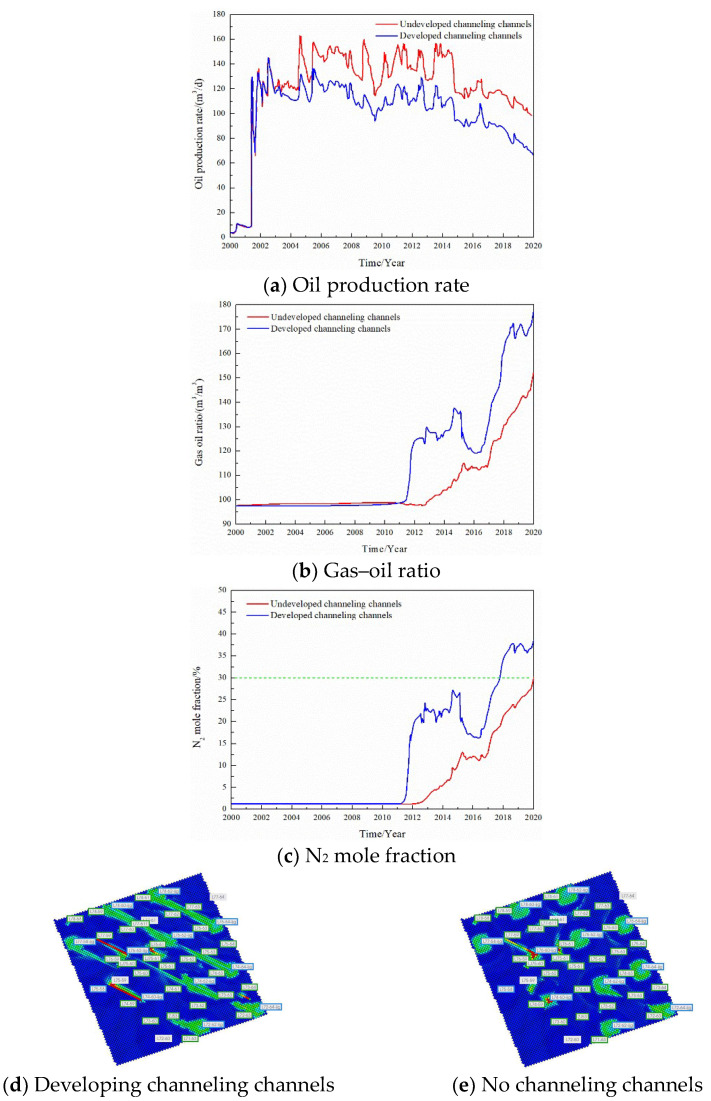
Effect of the channeling channel on the dynamic production performance.

By changing the development degree of the channeling channels, gas injection rate, and injection mode, the influence of the geological and dynamics production parameters on the gas channeling characteristics is studied. We are adjusting the distribution of high-permeability channels in the digital-analogue model of the target block and comparing the oil production rate, gas–oil ratio, and N_2_ mole fraction with and without channeling channels. Figure 7 shows that the development of channeling channels is the main factor affecting gas channeling. The natural and artificial fractures easily cause rapid gas channeling and result in an insufficient sweep volume. Therefore, there is a sharp decrease in the oil production rate and an increase in the gas–oil ratio when channeling channels are developed in oil reservoirs.

**Figure 8:**
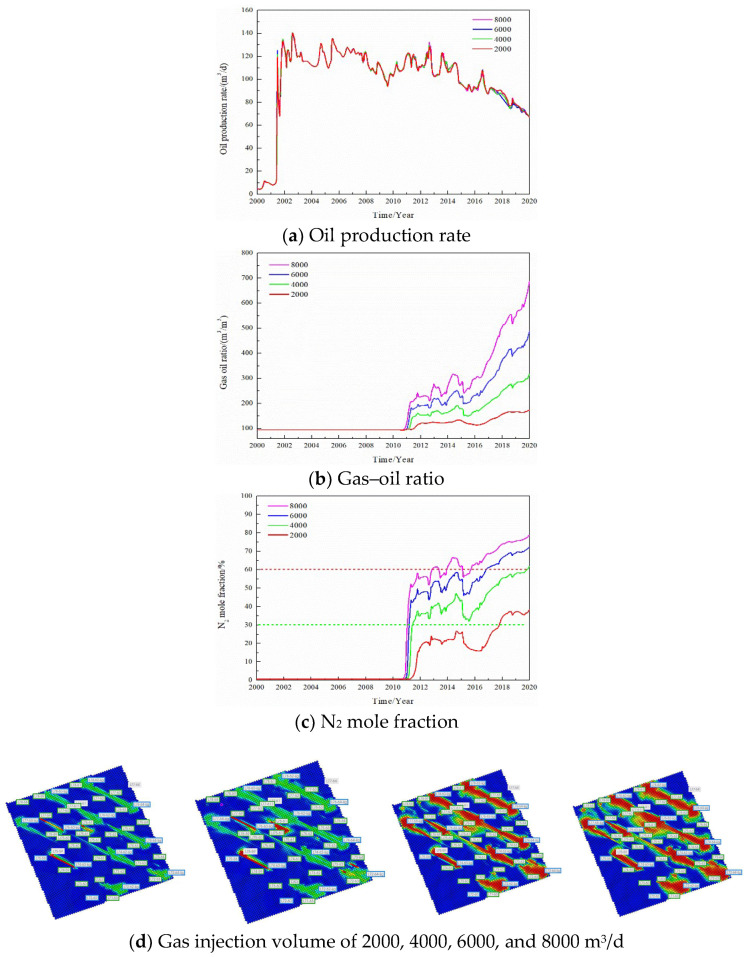
Effect of the gas injection volume on the dynamic production performance.

The gas injection rate was changed and the gas channeling laws were compared when the gas injection rate of a single well was 2000, 4000, 6000, and 8000 m^3^/d, respectively; the results are seen in Figure 8. The gas injection rate is one of the factors affecting gas channeling. When the gas injection rate of a single well increased from 2000 to 8000 m^3^/d, the oil production rate did not change significantly. However, the gas–oil ratio and the N_2_ mole fraction in the produced gas increased significantly, and the gas channeling became more serious. The gas channeling time was reduced from 7 to 1.5 years, the injected gas made serious channeling, and the utilization rate was significantly reduced. After increasing the gas injection rate, the volume swept by N_2_ in the reservoir did not increase and was still confined to the vicinity of the channeling channel, which cannot improve the gas injection development effect.

**Figure 9:**
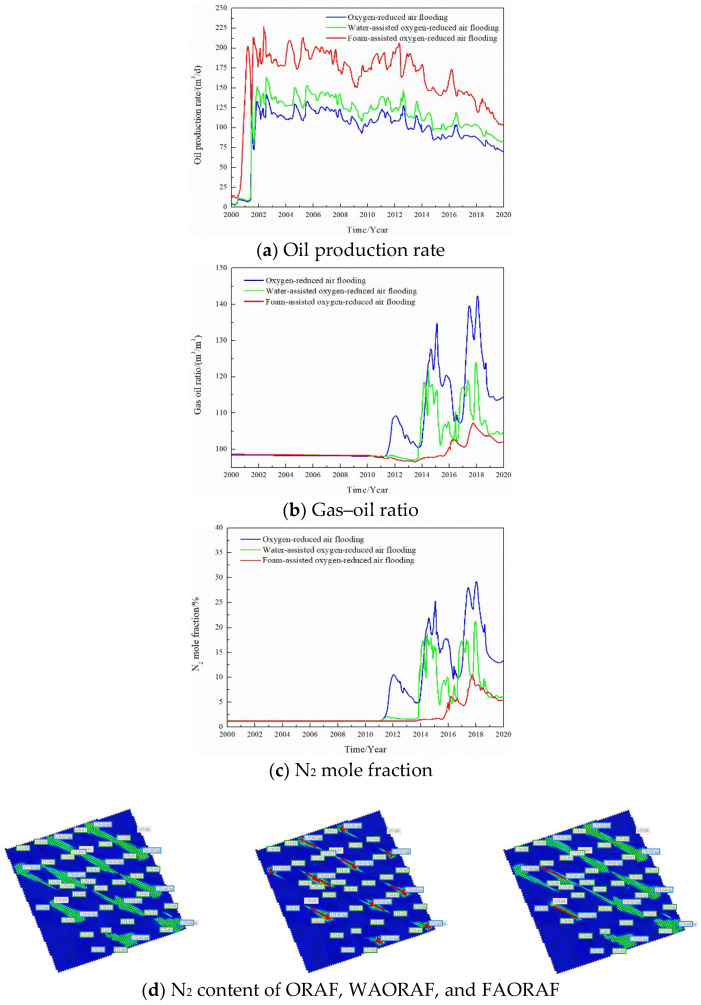
Effect of the gas injection mode on the dynamic production performance.

The gas injection method was changed, and the gas channeling laws of a single well at constant gas injection, water-assisted oxygen reduced air flooding (WAORAF), and FAORAF stages were comparatively analyzed. Figure 9 shows that, compared with continuous gas injection and water-assisted gas injection, FAORAF can significantly reduce the gas–oil ratio, increase the oil production rate, and inhibit gas channeling.

### 3.4. Static Performance of the Plugging System

**Figure 10:**
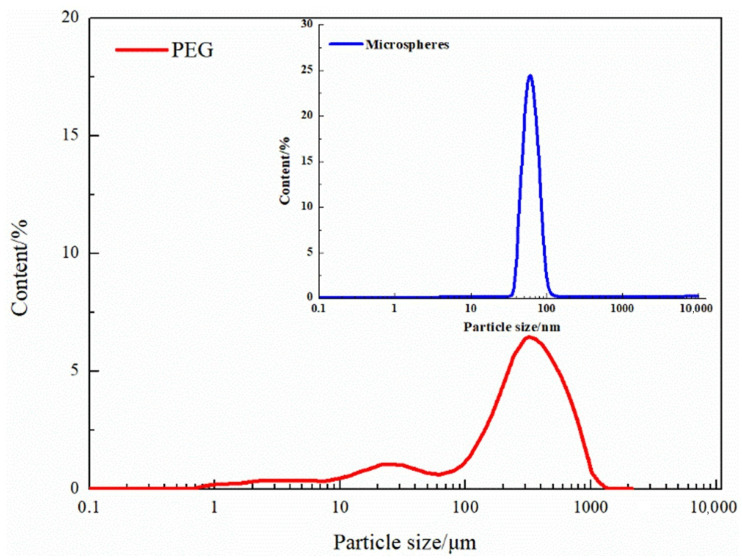
Particle size distribution diagram of the microspheres and PEG.

The particle size distribution of the microspheres and PEG is shown in Figure 10, and the test results of the hydration expansion performance are shown in Table 4. The particle size of the microspheres is mainly addressed between 37.84 and 105.709 nm, and the median size is 61.93 nm. The microspheres have a strong hydration expansion performance within 5 days, reaching more than 200 times. It became weak after 10 days, hydrolysis occurred after 20 days, and the particle size gradually decreased. The particle size of the PEG is mainly distributed between 203.6 and 468.4 μm, and the median particle size is 237.8 μm. The PEG hydration expansion ratio is 1.23–1.38 with 5–55 days and has better plugging stability. Compared to the microspheres, the PEG has a larger particle size (203.6–468.4 μm). Additionally, the PEG exhibits stable hydration swelling performance, which is suitable for blocking high permeability channels.

**Figure 11:**
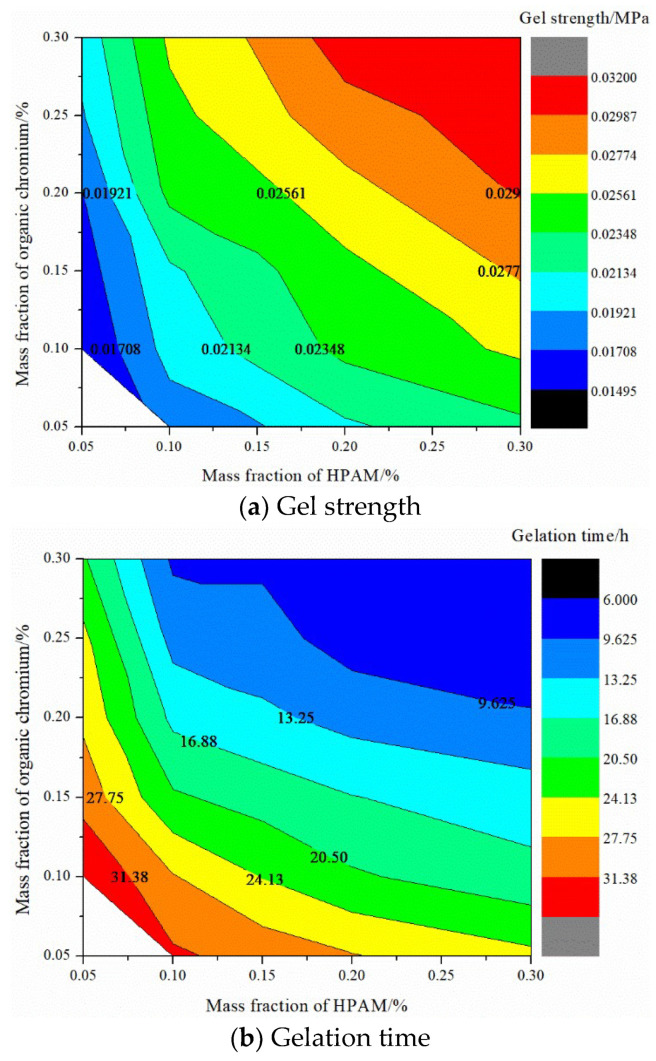
Contour diagrams of the static performance of organic chromium gel.

Under 55 °C, the viscosity of HPAM with different mass fractions is, in order, 5.8 mPa·s (0.05%) > 15 mPa·s (0.1%) > 222.6 mPa·s (0.3%) > 476 mPa·s (0.5%) > 1500 mPa·s (1%). Formation water was used to prepare the HPAM solution, and varying mass fractions of organic chromium cross-linking agents were added The gel strength and gelation time were obtained, as shown in Figure 11. The forming time of the gel is shortened with the polymer and cross-linker mass fraction, and the gel strength is increased. The gel formation time can be adjusted between 6–35 h, and the gel strength is 0.017–0.032 MPa. Considering the fluidity of the sealing and channeling system, the recommended formulation of the gel system is 0.1% HPAM + 0.1% organic chromium cross-linking agent to enhance the injectability, migration, and sealing performance of the gel in low-permeability reservoirs.

Taking dehydration time as the evaluation index, the stability performance of the gel system prepared with different concentrations of the organic chromium cross-linking agent is evaluated. A gel system (0.1% HPAM, 0.1% and 0.2% organic chromium cross-linking agent) of 15 mL each is prepared, and the gel system is placed at a reservoir temperature of 55 °C after gelation to compare the amount of water released from the gel system and the gel strength. Table 5 shows the stability evaluation results of the organic chromium gel and the nano-SiO_2_ organic chromium gel. The precipitation water volume of the gel increased with time, and the strength of the gel decreased. With the increase in the crosslinking agent concentration, the amount of precipitation water in the gel decreased, and the strength in the gel increased. Since the preferred gel system is a weak fluidity gel system, the breaking time is only 18 days. Therefore, for this gel, there is a shortcoming of a short stable period, which needs to be considered in the follow-up research process.

**Figure 12:**
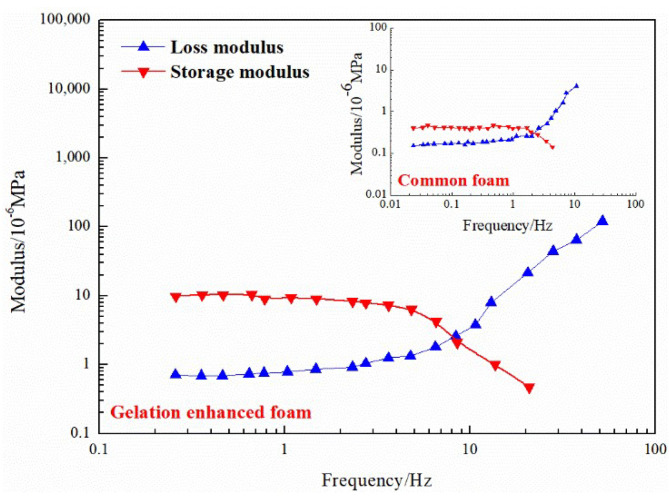
Viscoelasticity comparison: common foam and gel-enhanced foam.

Using formation water to prepare a 0.1% HPAM solution and adding 0.1% organic chromium cross-linking agent and different concentrations of nano-SiO_2_, the breakthrough vacuum degree of the nano-SiO_2_ organic chromium gel was measured. The breakthrough vacuum degree under different nano-SiO_2_ concentrations is, in order, 0.029 MPa (0.1%) > 0.028 MPa (0.05%) > 0.027 MPa (0.15%) > 0.026 MPa (0.20%) > 0.02 MPa (0). When the concentration of nano-SiO_2_ is greater than 0.1%, the binding structure of the colloid and nanoparticles is too loose. Additionally, the excessive concentration of the particles causes sedimentation and affects the performance of the gel [29]. The concentration of nano-SiO_2_ is 0.05–0.1%, which ensures the strength and fluidity of the gel. Figure 12 compares the viscoelastic properties of the ordinary foam and the gel-enhanced foam. The addition of gel increases the viscosity of the liquid phase and strengthens the mechanical strength of the foam liquid film. The analysis shows that the gel foam exhibits the characteristics of water-based foam before freezing and the characteristics of elastic gel after freezing. The enhanced foam with gel uses gel as the external phase, which has better stability than ordinary foam with water as the external phase and has a longer validity period for water blocking. The surface gel film of the jelly foam increases the elasticity of the liquid film, and the gas–liquid two-phase system increases the apparent viscosity so that the gel foam profile control system exhibits good viscoelasticity. The integrated gel foam has both the Jiamin effect of foam and the selective sealing effect of gel, which is more suitable for the deep sealing of high-permeability layers.

### 3.5. EOR Performance of the Plugging System

**Figure 13:**
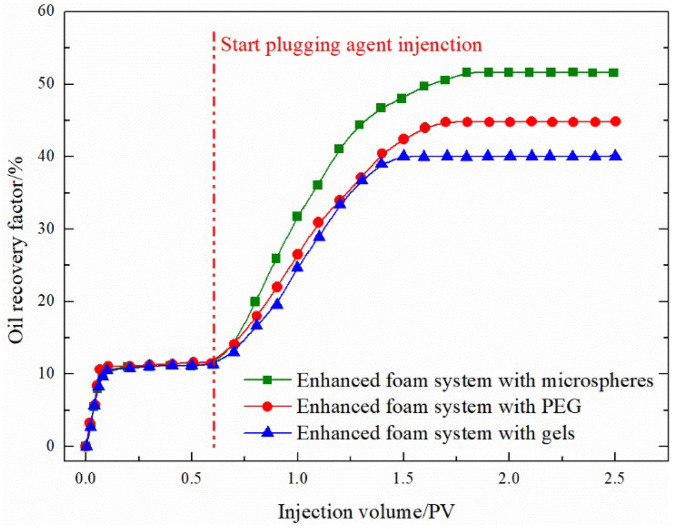
EOR experiment with a permeability ratio of 12.

For a higher permeability ratio of 12, the final gas-flooding recovery degree with a foam-assisted plugging agent is greater than 30%, as shown in Figure 13. For such high-level and poor reservoirs, plugging with a foam auxiliary plugging agent can better achieve the plugging effect such that the gas flooding recovery rate can reach more than 30%. The main reason for this is that the mobility of the foam flooding system improves the mobility ratio of the displacing phase and the displaced phase, and its Jamin effect can block large pores and selectively block oil and water. The surfactant in the system can reduce the oil–water interfacial tension and improve the oil washing efficiency [30,31].

**Figure 14:**
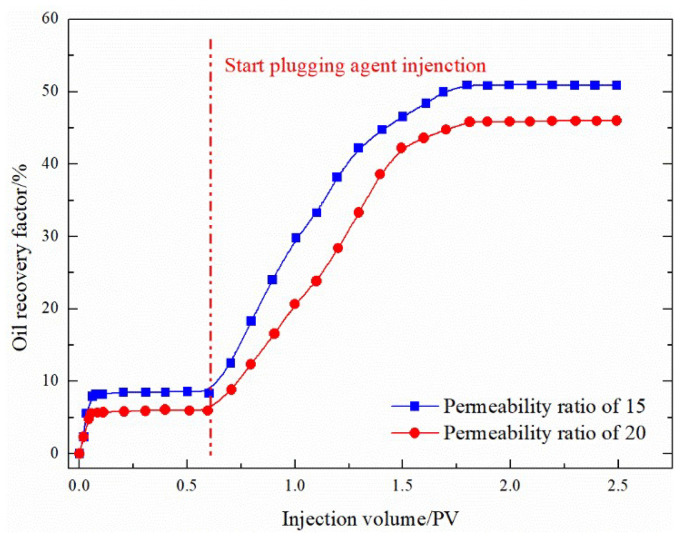
EOR experiment with a permeability ratio of 15 and 20.

In particular, for gel foam, the gel used has weak fluidity, the reinforced foam formed has strong fluidity, and the recovery rate of auxiliary gas flooding can reach more than 50%, so the plugging agent can better plug higher grade reservoirs. To study whether this movable gel foam can better seal high-level differential reservoirs, the physical model permeability difference is increased to 15 and 20 and the sealing effect of movable gel foam-assisted gas flooding on reservoirs with channeling channels is examined. As shown in Figure 14, movable gel foam-assisted gas flooding is suitable for reservoirs with a strong development of channeling channels, and the enhanced oil recovery rate for the differential 20 model is still up to 30%. The movable gel foam system is suitable for reservoirs with developed channeling channels and large permeability gradients.

**Figure 15:**
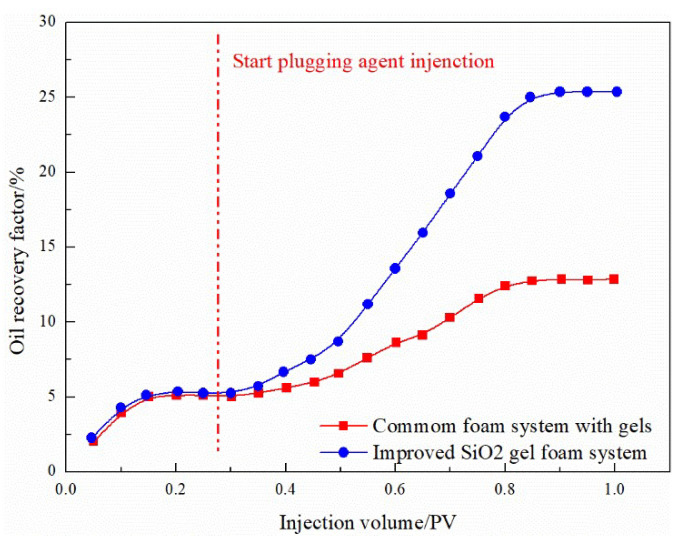
EOR experiment in the split core model.

Due to the presence of microfractures and a strong heterogeneity in the oil reservoir, it is recommended to use weak fluid gel foam for plugging to prevent gas channeling. However, the stability of the present gel foam plugging system is poor. To improve the system’s stability, nano-SiO_2_ is added to it, and the performance of the new gel foam system has been examined, as shown in Figure 15. The experimental results of simulated fractured reservoirs show that, after 0.1 PV gas injection, the oil recovery factor reached a steady state, which is 5.08%. After injecting a 0.2 PV ordinary gel foam system, the final recovery rate reached 12.86%, an increase of 7.78%. The ordinary gel foam system has a particular plugging effect on such fractured reservoirs, whereas, after injecting the 0.2 PV improved nano-SiO_2_ gel-enhanced foam system, the final recovery rate reached 25.24%, increasing by 20%. The new gel-enhanced foam system has a considerable plugging effect on fractured reservoirs. The analysis shows that, due to the addition of nano-SiO_2_ to the reinforced gel foam, the hydroxyl groups on the surface of the nanoparticles have the function of locking water, thereby prolonging the dehydration time of the gel and enhancing the stability of the gel foam system [32].

## 4. Conclusions

The conclusions of this work are as follows.

(1) Compared to water flooding, the channeling channels mainly develop along NE 60–70°, and FAORAF can significantly reduce gas channeling. Natural and artificial fractures are the main factors causing gas channeling, followed by the injection method and gas injection rate. Gas channeling tends to occur when the N_2_ mole fraction of the produced gas is greater than 60% and the gas–oil ratio is higher than 400 m^3^·t^−1^.
(2) Under the premise of the injection and migration efficiency, the optimal gel system is a 0.1% HPAM + 0.1% organic chromium crosslinking agent. The addition of gel increases the viscosity of the liquid phase and strengthens the mechanical strength of the foam liquid film.
(3) At a permeability ratio of 12, the recovery factors of the binary plugging systems composed of microspheres, PEG, and gel combined with foam were 40.89%, 45.85%, and 53.33%, respectively. Compared with the original system, the recovery rate of the improved ternary nano-SiO_2_ gelation foam system after 15 days of ageing using the core splitting test during the FAORAF process was 25.24%, an increase of 12.38%.

## Figures and Tables

**Table 1:** The ion composition of formation water in oil reservoirs.

Component	Cl^−^	HCO_3_^−^	Ca^2+^	Mg^2+^	SO_4_^2−^	Na^+^ + K^+^	Ba^2+^ + Sr^2+^	Total
Content/(mg·L^−1^)	13,985.025	54.918	415.024	137.503	2259.931	9438.953	2.146	26,293.68

**Table 2:** Parameters of the twin-tube model of the sand pack.

Binary Blocking System	Permeability Ratio	Pore Volume /mL	Permeability /(10^ −3^ μm^2^)
Enhanced foam with microspheres	12	77	105.4
		67.4	8.5
Enhanced foam with PEG	12	76.5	114.3
		68.2	9.3
Enhanced foam with gels	12	78.0	121.3
		69.0	9.7
	15	80.3	135.2
		67.5	8.9
	20	82.8	184.1
		68.0	9.1

**Table 3:** Classification standard of gas channeling in the FAORAF stage.

Number	Stage	N_2_ Mole Fraction/%	Change Features of N_2_	Gas–Oil Ratio/(m^3^·t^−1^)	Oil Production Characteristics
Ⅰ	No gas		Stable	<110	Increasing
Ⅱ	Gas front appearing	<30	Increasing	110–150	Stable
Ⅲ	Gas breakthrough	30–60	Increasing rapidly	150–400	Decreasing
Ⅳ	Gas channeling	>60	Stable	≥400	Decreasing

**Table 4:** Hydration and expansion performance of the microspheres and PEG.

Samples	Ageing Time/d	Size/μm	Media Size/μm	Expansion Times
Microspheres	5	13.34–20.39	12.95	209.11
	10	12.09–24.10	11.62	187.63
	20	2.678–4.618	2.501	40.38
	30	0.274–0.404	0.313	5.05
	55	0.122–0.190	0.1375	2.22
**Samples**	**Ageing Time/d**	**Size/nm**	**Media Size/nm**	**Expansion Times**
PEG	5	196.3–436.7	293.5	1.23
	10	196.3–436.6	287.3	1.21
	20	208–472.5	298.5	1.26
	30	201.7–442.6	308.4	1.30
	55	236.7–479.5	327.3	1.38

**Table 5:** Stability evaluation of the gel and nano-SiO_2_ gel.

Time		7 Days		15 Days		30 Days	
**Concentration of Crosslinker/Nano-SiO_2_**		**Separation of Water/mL**	**Breakthrough Vacuum/MPa**	**Separation of Water/mL**	**Breakthrough Vacuum/MPa**	**Separation of Water/mL**	**Breakthrough Vacuum/MPa**
Organic chromium gel	0.1%	Unapparent	0.023	3.4	0.011	Gel breaking	/
	0.2%	Unapparent	0.025	1.8	0.022	2.2	0.02
Nano-SiO_2_ organic chromium gel	0	Unapparent	0.023	3.4	0.011	Gel breaking	/
	0.05%	Unapparent	0.025	0.24	0.020	2.9	0.012
	0.1%	Unapparent	0.028	0.22	0.025	2.0	0.021

## Data Availability

Not applicable.
